# 3D gadolinium-enhanced high-resolution near-isotropic pancreatic imaging at 3.0-T MR using deep-learning reconstruction

**DOI:** 10.1186/s13244-025-02066-7

**Published:** 2025-09-24

**Authors:** Sylvie Guan, Julie Poujol, Elodie Gouhier, Caroline Touloupas, Alexandre Delpla, Isabelle Boulay-Coletta, Marc Zins

**Affiliations:** 1 Department of Medical Imaging, Saint Joseph Hospital, Paris, France; 2 Department of Medical Imaging, Rothschild Foundation Hospital, Paris, France; 3 MR Clinical Research, GE HealthCare, Buc, France; 4 Department of Medical Imaging, Pitié-Salpêtrière Hospital, Paris, France

**Keywords:** Pancreas, Magnetic resonance imaging, Deep learning, Image reconstruction, High-resolution

## Abstract

**Objectives:**

To compare overall image quality, lesion conspicuity and detectability on 3D-T1w-GRE arterial phase high-resolution MR images with deep learning reconstruction (3D-DLR) against standard-of-care reconstruction (SOC-Recon) in patients with suspected pancreatic disease.

**Materials and methods:**

Patients who underwent a pancreatic MR exam with a high-resolution 3D-T1w-GRE arterial phase acquisition on a 3.0-T MR system between December 2021 and June 2022 in our center were retrospectively included. A new deep learning-based reconstruction algorithm (3D-DLR) was used to additionally reconstruct arterial phase images. Two radiologists blinded to the reconstruction type assessed images for image quality, artifacts and lesion conspicuity using a Likert scale and counted the lesions. Signal-to-noise ratio and lesion contrast-to-noise ratio were calculated for each reconstruction. Quantitative data were evaluated using paired *t*-tests. Ordinal data such as image quality, artifacts and lesions conspicuity were analyzed using paired-Wilcoxon tests. Interobserver agreement for image quality and artifact assessment was evaluated using Cohen’s kappa.

**Results:**

Thirty-two patients (mean age 62 years ± 12, 16 female) were included. 3D-DLR significantly improved SNR for each pancreatic segment and lesion CNR compared to SOC-Recon (*p* < 0.01), and demonstrated significantly higher average image quality score (3.34 vs 2.68, *p* < 0.01). 3D DLR also significantly reduced artifacts compared to SOC-Recon (*p* < 0.01) for one radiologist.

3D-DLR exhibited significantly higher average lesion conspicuity (2.30 vs 1.85, *p* < 0.01). The sensitivity was increased with 3D-DLR compared to SOC-Recon for both reader 1 and reader 2 (1 vs 0.88 and 0.88 vs 0.83, *p* = 0.62 for both results).

**Conclusion:**

3D-DLR images demonstrated higher overall image quality, leading to better lesion conspicuity.

**Critical relevance statement:**

3D deep learning reconstruction can be applied to gadolinium-enhanced pancreatic 3D-T1w arterial phase high-resolution images without additional acquisition time to further improve image quality and lesion conspicuity.

**Key Points:**

- 3D DLR has not yet been applied to pancreatic MRI high-resolution sequences.
- This method improves SNR, CNR, and overall 3D T1w arterial pancreatic image quality.
- Enhanced lesion conspicuity may improve pancreatic lesion detectability.

**Graphical Abstract:**

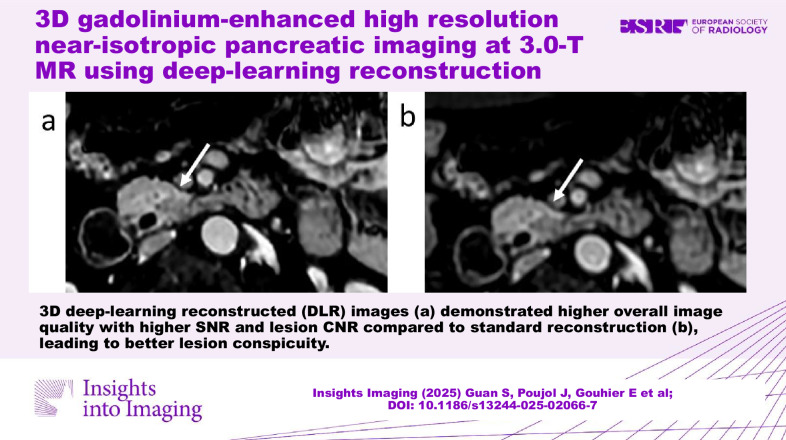

## Introduction

MRI has evolved into an essential modality in pancreatic imaging, complementing Computed Tomography (CT) [1]. CT is currently the primary choice for assessing pancreatic disease due to its excellent spatial resolution and accessibility. Despite its lower spatial resolution and its susceptibility to motion artifacts, MRI offers the advantage of being neither invasive nor irradiating, alongside superior contrast resolution, improving lesion characterization [2, 3]. Consequently, it is extensively used at diagnosis as a problem-solving tool and during follow-up routine.

The past decade has witnessed a surge in technical MR innovations ranging from hardware improvements such as high-density coils to software developments involving parallel imaging and compressed sensing. These advancements have allowed for faster examination times with improved spatial resolution and reduced noise while maintaining good image quality levels [4, 5].

Studies have shown that the 3D T1w Gradient Echo (GRE) arterial phase sequence with fat suppression offers the best detectability and conspicuity for pancreatic adenocarcinoma [6, 7]. Parallel imaging and compressed sensing have permitted to further improve this sequence, enabling near-isotropic spatial resolution within an acceptable acquisition duration [8, 9]. This allows reformatting of the 3D images in all planes without losing detail, improving analysis of lesions and their anatomical relations. However, the use of a high acceleration factor to acquire a high-resolution image within a fixed scan time may cause a decrease in SNR and generate additional noise and artifacts [4, 10].

The latest breakthrough in MR technology has been the development of Deep Learning Reconstruction (DLR) algorithms aimed at improving image quality and reducing acquisition durations when coupled with compressed sensing and parallel imaging [11, 12]. Various DLR implementations for upper abdominal imaging have been developed for 2D fast spin-echo (FSE) sequences [13, 14] and diffusion weighted-imaging (DWI) sequences [15, 16]. More recently, DLR has been adapted in 3D sequences such as T2w MR cholangiopancreatography (MRCP) sequences [17–19], and contrast-enhanced T1w GRE sequences in MR enterography [20]. Deep learning reconstruction has already been applied in pancreatic imaging on 3D T1w GRE contrast-enhanced images for super-resolution reconstruction with the aim of shortening acquisition times [21–23]. In those last studies, all acquisitions had been performed at 1.5 T with a 2-point DIXON technique for fat suppression.

The objective of this study was to evaluate at 3.0 T the impact of using a deep learning reconstruction algorithm applied to an arterial phase 3D T1w GRE Fat Saturated high-resolution sequence in comparison to the standard-of-care reconstruction algorithm. Based on previous results, we hypothesized that the 3D T1w GRE high-resolution arterial sequence with deep-learning reconstruction would demonstrate better image quality, lesion conspicuity and detectability with similar interobserver agreement compared to standard of care reconstruction.

## Materials and methods

### Patient population

This single-center retrospective study received approval from our institutional review board (GERM IRB 00012157). Written informed consent was waived, but a non-opposition form was sent to all patients. The inclusion criteria were patients who underwent a standard pancreas MR examination with gadolinium contrast injection at our institution on a 3.0-T MR system.

**Fig. 1:**
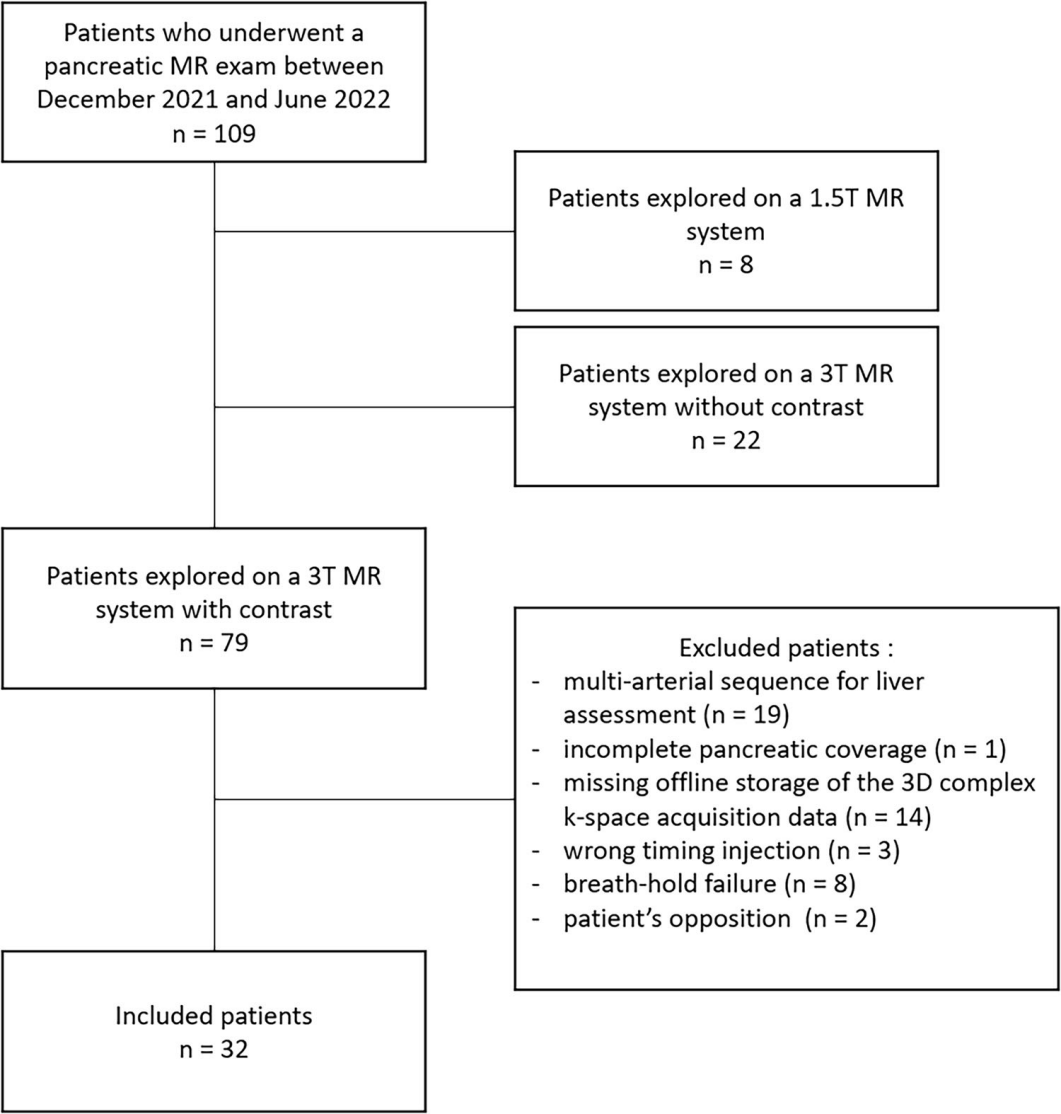
Flowchart of the study

Between December 2021 and June 2022, a total of 109 patients consecutively underwent a pancreatic MR examination with contrast injection in our center. Eight patients were explored on a 1.5-T MR system. Of the 101 patients scanned on a 3.0-T MR system, 22 patients did not need a contrast injection. Thus, a total of 79 subjects were identified for the study. Nineteen patients underwent a multi-arterial sequence for liver assessment, 1 patient had incomplete pancreas coverage on the arterial phase acquisition, offline storage of the 3D complex k-space was not available to reconstruct for 14 patients, injection was wrongly timed for 3 patients, 8 patients were excluded because of breath-hold failure, and 2 patients were opposed to the study. Ultimately, 32 patients were included. The flowchart of the study is pictured in Fig. 1.

### MR protocol

All pancreas examinations were conducted on a 3.0-T whole-body clinical MR system (SIGNA Premier; GE HealthCare) using a dedicated 30-ch Anterior phased-array receiver coil combined with a 60-ch posterior phased-array receiver coil embedded in the MR table (GE HealthCare AIR^TM^ Coils). Respiratory belt, navigator or breath-hold techniques were used to ensure adequate image quality depending on the sequence type. Additionally, every patient received an intravenous injection of an antispasmodic agent (Glucagen; 1 mg/mL; Novo Nordisk) immediately before the examination to decrease bowel peristalsis.

The standard MR pancreas protocol performed in our center included several sequences before injection: a DWI sequence (three directions and 2 b-values; b = 50 s/mm² and b = 800 s/mm²), and T2 and T1-weighted sequences with and without fat suppression.

A gadolinium-based extracellular (Dotarem^®^; Guerbet or Gadovist^®^; Bayer) contrast agent was intravenously injected at 0.1 mmol/kg of body weight, followed by a 30 mL saline flush. Several breath-hold T1w Gradient Echo acquisitions were then acquired to image distinct circulatory phases (arterial (20 s), portal venous (60–70 s) and late phase (5 min)). Our sequence of interest was the axial T1-w Gradient Echo sequence centered on the pancreas during the arterial phase (Axial LAVA Fat Sat IV+), with an acquisition voxel size of 1.5 × 1.6 × 1.4 mm^3^ and a reconstructed voxel size of 0.4 × 0.4 × 0.7 mm^3^. More details about this sequence can be found in Table 1.

**Table 1:** Axial LAVA Fat Sat IV+ acquisition parameters

MR parameters	Axial LAVA Fat Sat IV+
Pulse type	3D GRE
TR/TE (ms)	3.2/1.4
Flip angle (degrees)	12
Saturation type	Adiabatic SPectral Inversion Recovery (ASPIR)
TI (ms)	23
Receiver bandwidth (kHz)	90.91
FOV (mm)	420
Phase FOV	0.8
Acquisition matrix (freq × phase)	280 × 256
Acquisition voxel size (mm)	1.5 × 1.6 × 1.4
Reconstructed voxel size (mm)	0.4 × 0.4 × 0.7
Number of averages	1
Parallel imaging	ARC
Phase direction reduction factor	2
Slice direction reduction factor	1.15
Compressed sensing factor	1.2
Acquisition time (s)	19

The MR examination ended with several MRCP sequences (3D, 2D, free breathing and/or breath hold depending on patient condition) to image pancreatic and biliary ducts when needed.

### Deep-learning reconstruction

A vendor-supplied prototype of the 3D AIR^TM^ Recon DL reconstruction algorithm (3D DLR) was used to reconstruct the arterial phase acquisition offline. Similar to the 2D AIR^TM^ Recon DL version [11], our 3D DLR prototype consists of a feed-forward deep Convolutional Neural Network (CNN) embedded in the MR image reconstruction path that generates enhanced images from complex-valued raw data. The deep-learning algorithm is designed and trained to reconstruct interpolated magnitude images with higher SNR and reduced truncation artifacts in all three directions. For SNR enhancement, the CNN does not directly generate high SNR images but rather estimates the image noise. Noise reduction is then chosen based on user preferences through a user-specified denoising level, a scalar parameter between 0% and 100%. This avoids blind-aggressive denoising by adding back a little controlled level of noise, making it appear more natural to the human eye. The noise reduction factor in our study was set to 75% based on previous experience with the 2D AIR^TM^ Recon DL version in our center and initial tests on 10 patients not included in the study. The network underwent training via a supervised learning approach using more than 10,000 pairs of near-perfect (high resolution, low noise) and typical (low resolution, noisy) images from a broad range of types. Image augmentations, including rotations, flips, intensity scaling, and additive noise, were used, ultimately resulting in an extremely large and diverse training database of 4.4 million unique images for added robustness [11]. Both 2D and 3D networks were trained in a similar way; however, the data used to train both algorithms were adapted for the unique acquisition methods used in 2D and 3D imaging.

### Image analysis

Images were independently reviewed on dedicated Picture Archiving and Communication System (PACS) workstations (Carestream, Philips) on diagnostic quality monitors.

Two radiologists (one senior radiologist with 30 years experience in abdominal imaging, reader 1, M.Z., and one junior with 4 years experience, reader 2, S.G.) independently reviewed the 3D T1w GRE arterial sequences, blinded to the reconstruction technique and patients’ clinical history. The radiologists performed the review in separate sessions to minimize the effect of fatigue and habits. In addition, the sequences were scrambled to reduce bias, meaning one session could contain a random number of standard and deep-learning reconstruction sets.

For lesion detection, the standard of reference was established based on a consensus review of all imaging studies dedicated to the pancreas (US, CT, PET-CT, EUS, MRI) performed by both radiologists after 1 month of wash-out period to reduce recall bias. For that purpose, all initially performed MR sequences were available for each patient, including the 3D T1w GRE HR arterial phase with standard reconstruction but excluding the deep-learning reconstruction.

Overall image quality was subjectively assessed on a 4-point Likert scale (1: non-diagnostic images, 2: poor or below average, 3: good or above average, 4: excellent). Artifacts assessment was also conducted on a 4-point Likert scale (1: non-diagnostic images due to major artifacts, 2: some diagnostic uncertainty due to moderate artifacts, 3: minor artifacts with no adverse effect for clinical use, 4: no artifacts). Pancreatic lesions were counted (up to 5), and the location of each lesion was recorded for each reconstruction technique. Overall lesion conspicuity was assessed on a 3-point Likert scale for the MR examinations containing at least one lesion (1: poor conspicuity, 2: average conspicuity, 3: excellent conspicuity).

The study coordinator (S.G.) performed the quantitative analysis of image quality by calculating the signal-to-noise ratio (SNR) in three pancreatic areas and contrast-to-noise ratio (CNR) between lesions and normal pancreatic parenchyma. Regions-of-interest (ROIs) of the same size and shape were drawn for each patient in head, body, and tail of the pancreas at the same location for both standard and deep learning reconstructions. For patients with identified lesions, ROIs of the same size and shape were also drawn inside the lesion (or the bigger one in case of several lesions) and in the adjacent parenchyma. Mean signal intensity was measured in each ROI, and the standard deviation of signal intensity with another ROI placed outside the patient near the skin was considered as noise (Supplemental Figures). The SNR in each pancreas location was calculated by dividing mean signal intensity by the noise. The CNR between a lesion and normal pancreas parenchyma was defined as the difference in the mean signal intensity of the lesion and the adjacent parenchyma divided by the noise.

### Statistical analysis

All continuous data were tested for normality before analysis using the Shapiro–Wilk test due to the small number of patients and were expressed as mean ± SD. Comparison of SNR and CNR between both reconstructions was performed using paired *t*-tests.

Qualitative scores (Overall image quality, Image Artifacts and Lesion conspicuity) were expressed as means and analyzed statistically using the Wilcoxon signed-rank paired test.

Interobserver agreements on image quality, presence of artifacts and lesion conspicuity between the two radiologists were evaluated for each sequence using the weighted Cohen kappa (κ) test. A κ value ≤ 0.20 indicated slight agreement; 0.21–0.40, fair agreement; 0.41–0.60, moderate agreement; 0.61–0.80, substantial agreement; and 0.81–0.99, almost perfect agreement.

Sensitivities and specificities for detecting at least one pancreatic lesion were compared for each reader using the McNemar Chi2 test.

All statistical tests were conducted at a significance level of *p* < 0.05. Statistical analyses were performed using R software.

## Results

### Patient characteristics

Thirty-two patients (mean age 62 years ± 12, 16 female) were included for subsequent deep learning image reconstruction and analysis. The study population characteristics are summarized in Table 2. The mean acquisition time (breath-hold duration) for the 3D GRE arterial phase was 22 s (range: 19–29 s). A total of 38 lesions were counted across 24 patients, with 8 patients not having any lesion.

**Table 2:** Patient characteristics and clinical indications

Characteristics	Value
Age (years)	62 ± 12 [29–82]
Sex	
Men	16 (50%)
Women	16 (50%)
BMI (kg/m²)	25.6 ± 4.6
Indication for MRI examination	
Cystic or solid pancreatic lesion detected on ultrasound or CT initial evaluation	11
IPMN follow-up with 1 or more lesion(s) > 3 cm	4
Chronic pancreatitis initial evaluation or follow-up	3
Acute pancreatitis etiologic assessment	6
Pancreatic neuroendocrine tumor follow-up	2
Pancreatic cyst (not IPMN) follow-up	3
Wirsung duct dilation	1
Pancreatic cancer screening in a high-risk patient	1
Pancreatic calcification assessment with Wirsung duct dilation	1

### Qualitative image analysis

Results on qualitative image analysis are summarized in Table 3. The overall image quality was significantly increased with 3D DLR compared to SOC-Recon for both reader 1 and reader 2 (3.23 vs 2.32 and 3.45 vs 3.03, respectively, *p* < 0.01). Artifacts were significantly reduced with 3D DLR for one reader (3.23 vs 2.81, *p* < 0.01) but not for the other (3.29 vs 3.19, *p* = 0.39).

**Table 3:** Evaluation of overall image quality, motion artifacts and lesion conspicuity on deep learning reconstructed images and standard reconstructed images by two independent readers

Variables	Reader 1			Reader 2		
	3D DLR	SOC Recon	*p*-value	3D DLR	SOC Recon	*p*-value
Overall image quality	3.23 ± 0.62	2.32 ± 0.65	< 0.001	3.45 ± 0.88	3.03 ± 0.54	< 0.01
Motion artifacts	3.23 ± 0.69	2.81 ± 0.79	< 0.01	3.29 ± 0.76	3.19 ± 0.60	0.39
Lesion conspicuity	2.39 ± 0.73	1.88 ± 0.60	< 0.01	2.18 ± 0.68	1.81 ± 0.61	< 0.01

**Fig. 2:**
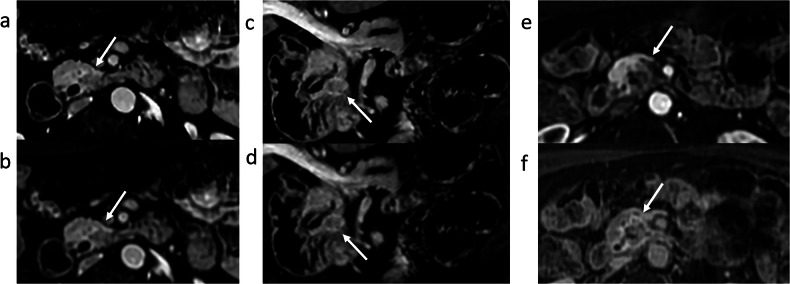
A 62-year-old man with a pancreatic head lesion detected on a CT scan performed for sigmoid tumor staging. The high-resolution DLR images (**a** axial plane, and **c** reconstructed coronal-oblique plane) show improved image quality with reduced noise compared to standard images (**b**, **d**) as well as sharper edges with better conspicuity of the lesion (arrows), which was suspicious for pancreatic adenocarcinoma, with a small mass effect on the pancreatic canal. A first EUS following MRI examination was negative. 16 months follow-up showing progression of lesion (**e**, **f**). Endoscopic ultrasound biopsies later confirmed pancreatic adenocarcinoma

**Fig. 3:**
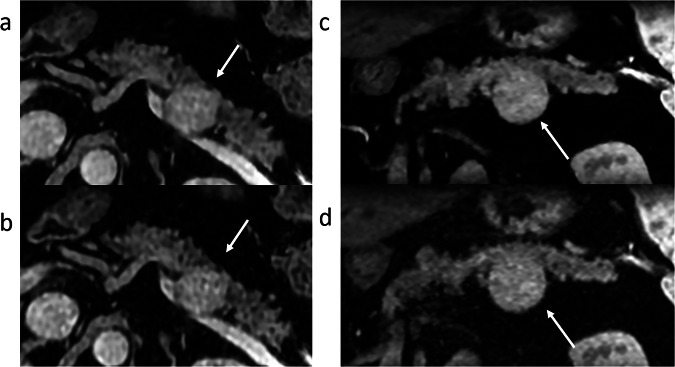
A 53-year-old man with a pancreatic tail mass detected on a CT scan. The high-resolution DLR images show better image quality with reduced noise, as well as higher contrast of the lesion (arrows) and improved edge delimitation (**a** axial plane, and **c** reconstructed coronal-oblique plane) compared to standard images (**b**, **d**). Final diagnosis was solid pseudopapillary tumor of the pancreas

**Fig. 4:**
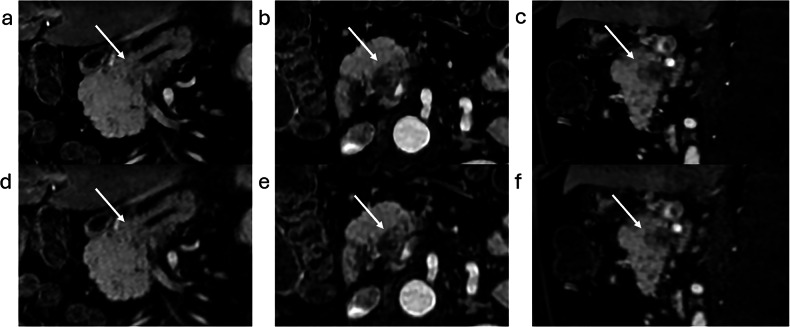
A 62-year-old man presenting with acute pancreatitis, with a pancreatic head mass detected on a CT scan. Multiplanar reformatted high-resolution DLR images (**a** coronal, **b** axial, **c** sagittal plane) show enhanced image quality with reduced noise and sharper edges of the lesion (arrows) compared to standard images (**d** coronal, **e** axial and **f** sagittal plane). Final diagnosis was pancreatic adenocarcinoma

Lesion conspicuity was significantly better using 3D DLR compared with SOC-Recon according to both readers (2.39 vs 1.88 and 2.20 vs 1.81, *p* < 0.01). Three examples are shown in Figs. 2, 3 and 4.

The interobserver agreement for overall image quality was slight with SOC-Recon (k = 0.09), and moderate with 3D DLR (k = 0.54). It was moderate for motion artifacts with SOC-Recon (k = 0.48), and substantial with 3D DLR (k = 0.69). It was moderate for lesion conspicuity for both SOC-Recon (k = 0.41) and 3D DLR (k = 0.29).

### Quantitative image analysis

**Fig. 5:**
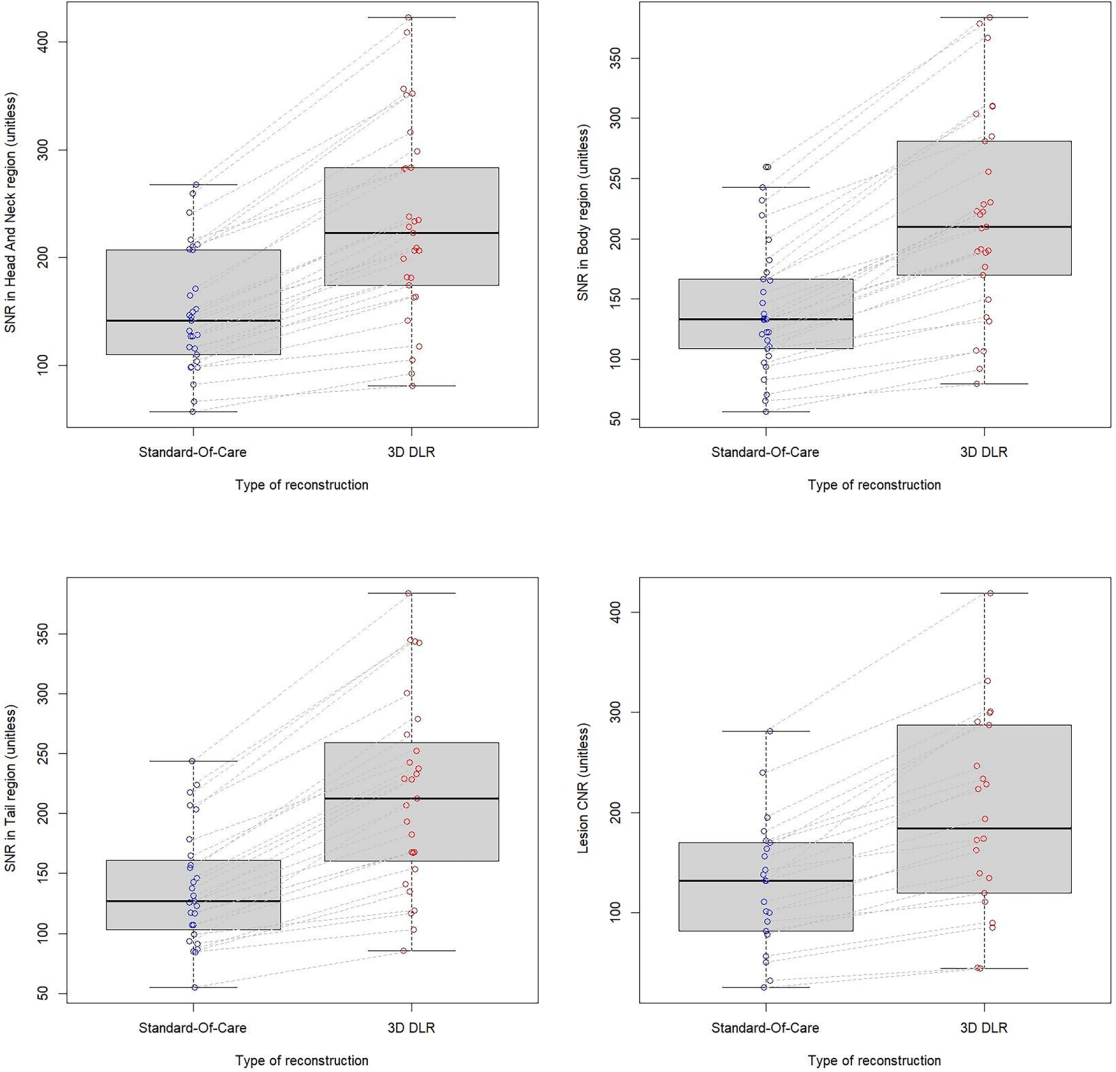
Boxplots of signal-to-noise ratio (SNR) and lesion contrast-to-noise ratio (CNR) between standard-of-care reconstruction (SOC) and 3D DLR reconstruction (3D DLR). 3D DLR systematically improves SNR in all pancreatic subparts and CNR of the detected lesions

3D DLR significantly improved signal-to-noise ratio for each pancreatic part compared to SOC-Recon (226.39 vs 147.93 for head and neck, 214.59 vs 139.19 for body, 199.32 vs 129.07 for tail, all *p* < 0.01). The contrast-to-noise ratio of detected lesions was also significantly improved with 3D DLR compared to SOC-Recon (12.60 vs 11.15, *p* < 0.01) (see Table 4 and Fig. 5).

**Table 4:** Comparison of signal-to-noise ratio (SNR) and lesion contrast-to-noise ratio (CNR) between standard-of-care reconstruction (SOC) and 3D DLR reconstruction (3D DLR)

Reconstruction type	SOC Recon			3D DLR			*p*-value
Pancreatic subpart	Head and neck	Body	Tail	Head and neck	Body	Tail	
SNR	147.93 ± 54.56	139.19 ± 51.27	129.07 ± 56.22	226.39 ± 88.61	214.59 ± 82.13	199.32 ± 91.02	< 0.01
Lesion CNR	11.15 ± 10.39			12.60 ± 12.38			< 0.01

### Diagnostic accuracy for lesion detection

Sensitivity was increased with 3D DLR compared to SOC-Recon for both reader 1 and reader 2 (1 vs 0.88 and 0.88 vs 0.83, *p* = 0.62 and *p* = 0.62, respectively), and specificity was lower with DLR for reader 1 (0.43 vs 0.57, *p* = 0.48 and 0.86 vs 0.86).

## Discussion

Our study showed that 3D DLR significantly improved pancreas Signal-to-Noise Ratio and lesion Contrast-to-Noise Ratio compared to standard-of-care reconstruction, resulting in higher lesion conspicuity with better overall image quality. Although the image quality is improved, the present study did not show the advantage of the algorithm in diagnostic performance with the current sample size.

There is limited research on how to achieve isotropic or near-isotropic 3D T1w GRE contrast-enhanced sequences in upper abdominal imaging. One study by Kim et al [8] showed that high in-plane spatial resolution images obtained with compressed sensing, covering both the liver and pancreas, demonstrated significantly increased organ edge sharpness compared to standard resolution imaging without increased motion artifacts but with increased noise, although it did not address lesion detection or conspicuity. Another study by Sun et al [24] focusing on the liver compared isotropic spatial resolution (1.1 mm^3^ voxel size) gadolinium-enhanced late phase 3D T1 GRE images acquired using a combination of compressed sensing and parallel imaging versus standard spatial resolution (0.9 × 0.9 × 4 mm^3^) obtained with parallel imaging only in patients with hepatocellular carcinoma. No significant difference in overall image quality was found in this study, but isotropic images showed better liver-to-lesion contrast despite more motion artifacts due to a longer acquisition time (16 s vs 12 s). SNR and lesion detectability were not evaluated. As described in both studies, compressed sensing helps to increase anatomical coverage and spatial resolution while keeping a short acquisition time for reasonable breath-holding durations and good image quality.

Previous research has highlighted the utility of deep learning applied to 3D gradient echo imaging of the pancreas on 1.5-T MR scanners. In a retrospective series of 50 patients with pancreatic lesions conducted by Chaika et al [22], deep learning was applied to already reconstructed images to enhance spatial resolution. The deep-learning processed 3D T1w GRE 2-points DIXON images showed superior lesion detectability, lower noise levels, enhanced sharpness and contrast of pancreatic edges and vessels, alongside increased diagnostic confidence compared to standard-of-care reconstruction. However, this study did not focus on lesion detection since included patients all presented at least one lesion and was conducted exclusively on 1.5-T MR systems with a voxel size of 1.19 × 1.19 × 3 mm^3^ size in contrast to our study, conducted at 3.0 T with a similar acquisition time but with a reconstructed voxel size of 0.4 × 0.4 × 0.7 mm^3^.

The DLR algorithm used in our study can also be applied to 3D T1w GRE sequences using near-isotropic spatial resolution in other areas of abdominal imaging, such as MR enterography in patients with Crohn’s disease [20]. In this study by Son et al, this application has demonstrated similar results with improved SNR, overall image quality, sharpness and contrast, along with decreased perceived motion artifacts and reduced noise compared to standard-of-care reconstruction, with a voxel size of 1.3 × 1.5 × 1.6 mm^3^. Low SNR is usually a limitation of high spatial resolution MR imaging. The DLR algorithm’s ability to denoise images and increase SNR was further demonstrated in our study, in which we used a higher spatial resolution coupled with a smaller imaging volume for pancreas imaging in comparison to the lower spatial resolution and larger field of view needed for MR enterography.

A recent study by Chaika et al [23] showed that prospectively using deep learning reconstruction (DLR) allowed for a significant reduction in acquisition time for T1-weighted volumetric interpolated breath-hold examination (VIBE) with Dixon fat suppression on 1.5-T scanners. This approach improved image quality, diagnostic confidence, and lesion conspicuity while reducing motion artifacts and noise. However, their method differs from ours in that it did not apply DLR to the arterial phase acquisition, and focused on shorter acquisition times rather than achieving high spatial near-isotropic resolution, with a reconstructed voxel size of 0.6 × 0.6 × 3 mm^3^. Additionally, they experienced minor aliasing artifacts potentially related to the partial Fourier setting, which is known to induce Gibbs ringing.

Future research could focus on developing deep-learning reconstruction algorithms specifically tailored to highly undersampled data, to produce highly accelerated sequences that are reconstructed without undersampling artifacts while simultaneously enhancing signal-to-noise ratio (SNR) and spatial resolution.

Our study had several limitations. First, it was a monocentric retrospective study conducted in a tertiary center, potentially introducing a selection bias. Second, it was conducted exclusively on 3.0-T MR scanners, limiting the generalizability of these findings given the wider prevalence of 1.5-T scanners. Third, the cohort was small, resulting in a low number of lesions and limited statistical power; however, we believe that increasing the cohort size would not alter our results. While the statistical outcomes for SNR and CNR might improve slightly, the detection of lesions would remain similar since the baseline sequence was finely optimized. Some patients diagnosed with pancreatic adenocarcinoma were not included in this study due to the use of thicker slices screening for liver metastases in the arterial phase. Finally, our 3D T1w GRE arterial phase sequence was relatively long with a 22-s acquisition mean time, which could limit the reproducibility for some challenging patients.

In conclusion, the results of this study demonstrate that deep-learning reconstruction can be used in 3D T1 GRE contrast-enhanced pancreatic imaging with near-isotropic spatial resolution to further improve image quality and SNR, along with reducing artifacts. It allows for high-quality 3D reformatted images and higher lesion CNR, resulting in better lesion conspicuity.

## Supplementary information

ELECTRONIC SUPPLEMENTARY MATERIAL

## Data Availability

Limited data available due to individual privacy. Please contact the corresponding author.

## Abbreviations

CNR: Contrast-to-noise ratio
DLR: Deep learning reconstruction
DWI: Diffusion weighted imaging
GRE: Gradient echo
MRCP: MR cholangiopancreatography
ROI: Regions-of-interest
SNR: Signal-to-noise ratio
SOC-Recon: Standard-of-care reconstruction
T1w: T1-weighted
