# Supplementary material for: 3D gadolinium-enhanced high-resolution near-isotropic pancreatic imaging at 3.0-T MR using deep-learning reconstruction
Source: Insights Imaging. 2025 Sep 24;16:204. doi: 10.1186/s13244-025-02066-7 (PMC12460215; doi:10.1186/s13244-025-02066-7)
Supplement: Supplementary file 1 — ELECTRONIC SUPPLEMENTARY MATERIAL [file 13244_2025_2066_MOESM1_ESM.pdf]

3D gadolinium-enhanced high resolution near-isotropic pancreatic imaging at 3T MR using deep-learning reconstruction

ELECTRONIC SUPPLEMENTARY MATERIAL

Insights Imaging (2025) Guan S, Poujol J, Gouhier E, et al.

Supplementary figure 1 :
Placement of ROI for SNR calculation

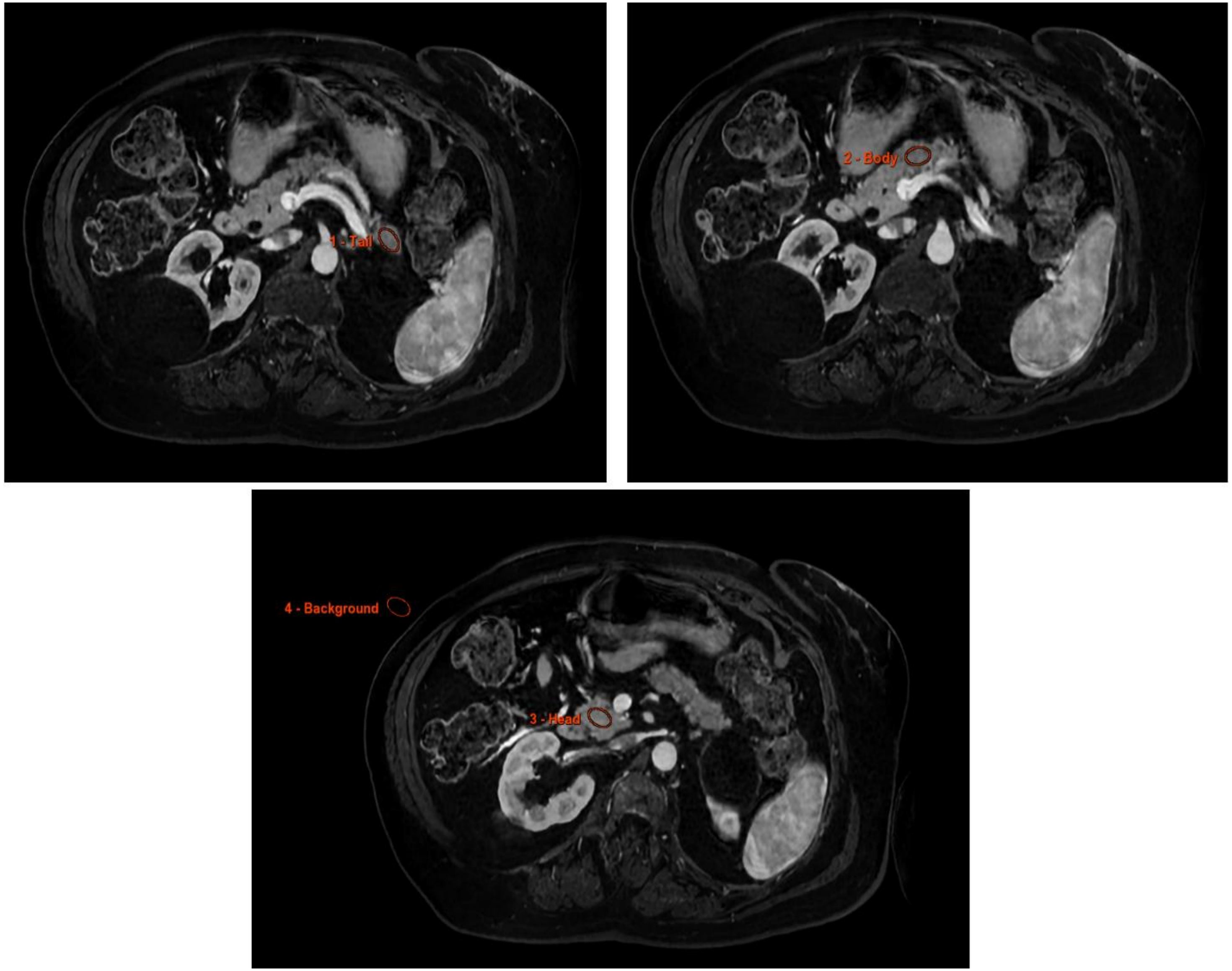

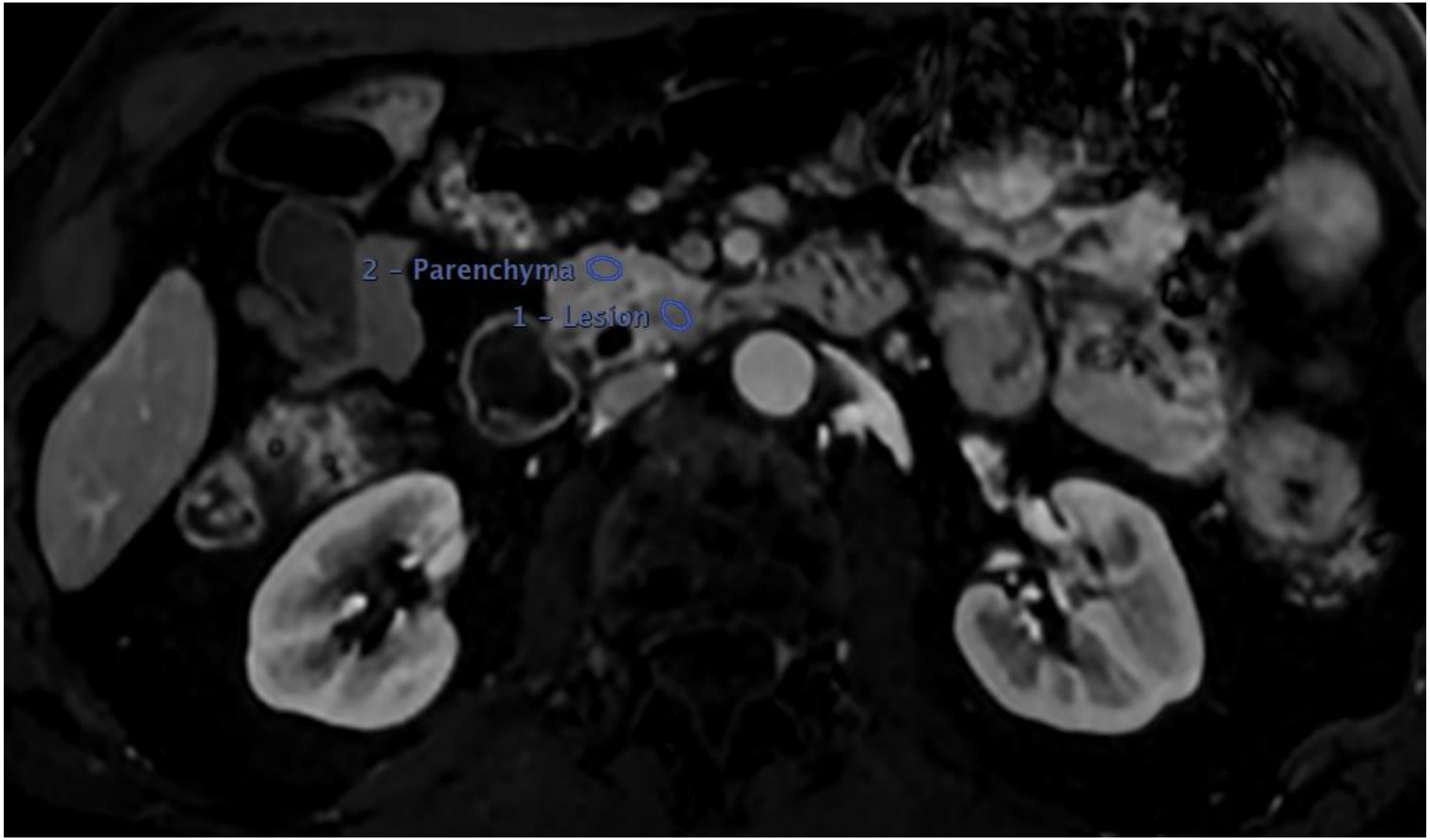

Supplementary figure 2 : Placement of ROI for lesion CNR calculation
